# Residents' preferences for urban agriculture in Shanghai

**DOI:** 10.1016/j.heliyon.2024.e30974

**Published:** 2024-05-09

**Authors:** Shengyi Du, Katsuya Tanaka

**Affiliations:** a Graduate School of Economics, Doctorate Program, Shiga University, Japan; b Faculty of Economics / Research Center for Sustainability and Environment, Shiga University, Japan

**Keywords:** China, Choice experiment, Food safety, Organic, Urban agriculture

## Abstract

Although urban agriculture (UA) has been gaining greater attention as part of an effort toward sustainable urban development in China, empirical knowledge is limited. We conducted a discrete choice experiment with 756 residents of Shanghai. Overall, the results indicate that respondents supported UA as they tended to favor one of the UA scenarios presented. Residents prefer UA facilities that utilize environmentally friendly production, offer farming activities, and are equipped with dining facilities. Regularly scheduled educational activities hosted by UA facilities are preferred to those on an irregular basis. In terms of location, residents prefer UA facilities that are close by but do not necessarily want UA facilities to be right in front of them. There is a stronger preference for UA among highly educated populations, those with primary and secondary school students, and those with agricultural work experience. Additionally, various UA attributes affect residents' preferences; overall, there is a high degree of homogeneity in residents’ preferences for various UA attributes. The marginal willingness to pay value is slightly higher than expected among the estimation results. There are three reasons for this outcome: the characteristics of UA as a recreational facility in China, the relatively high price level in Shanghai, and the impact of the COVID-19 pandemic.

## 1 Introduction

The rapid expansion of urban areas in both developing and developed countries poses a serious threat to global warming and biodiversity [1]. Although cities represent only 2 % of the world's land mass, urban greenhouse gas emissions account for 70 % of total human emissions [2]. In terms of biodiversity, habitat loss due to urban growth was 190,000 square kilometers between 1992 and 2000, with a projected loss of 290,000 square kilometers between 2000 and 2030 [3,4]. Furthermore, soil sealing,^1^ stormwater runoff, groundwater replenishment blockages, and water contamination in urban areas are becoming increasingly serious [[5], [6], [7]].

Urban agriculture (UA) has garnered attention as a viable effort to mitigate these problems. UA refers to agricultural actions that occur in cities or metropolitan areas and their surrounding zones [8,9]. In addition to the food supply, UA has diverse functions such as improving environmental issues, providing green space, and education [9].

UA significantly improves the environment. Many UA facilities focus on arboricultural cultivation. Nowak and Crane (2000) observed that arboriculture in urban areas can reduce ozone, sulfur dioxide, and PM10 by 15 %, 14 %, and 13 %, respectively, within a maximum of 1 h [10]. Additionally, Lin et al. (2017) revealed an increase in plant diversity due to the implementation of UA increase in animal diversity, especially in arthropods [11]. The same effects can also be seen in other cases, such as the Agrotechnology Parks in Singapore, Green Belt in the United Kingdom, and urban agricultural projects in Cuba [12].

There is much research on people who regularly engage in UA farming. These studies have revealed the following: a higher percentage of UA participants are non-professional farmers who are more motivated to farm [13], farmers who operate tourist-type farms have a higher awareness of environmental conservation [14], hobbies and food security are the main motivations for people to engage in UA [15], and welfare policies significantly impact their motivation [16].

Although there are relatively few surveys on the preferences of urban residents, they reflect, to a certain extent, the attitudes of residents toward UA. Urban residents as consumers are the support base for sustainable UA management, and most of them have positive attitudes. However, their awareness of UA is relatively low due to a lack of information provision. Furthermore, food safety and dietary improvements related to UA have attracted the most interest, but residents’ willingness to purchase UA products is almost the same as that for general products [17,18].

There is a lack of research on urban residents’ preferences in prior studies, with China as the study target. Most studies were case studies of specific areas or policy analyses. Yuan and Chen (2015) and Jin (2020) have pointed out the rapid urbanization of rural areas and the serious decline of farmland due to the leasing of farmland (use rights) in the peri-urban zone [19,20]. At the same time, they emphasized the need for UA and hoped to solve the problem of farmland loss. Wang (2021) also highlighted that China does not have its own UA implementation standards, and land use regulations regarding UA are unclear [21].

Notably, the positioning of UA in the West and China is different. In the West, farming behavior is required from the residents. They rent vacant land in urban areas and farm freely (e.g., “Kleingarten” in Germany) or participate in regular farming activities as volunteers in management-style farms (e.g., Zenger Farm in Portland). In China, UA is expected to be recreational, and residents participate as consumers. Farming experience and educational activities are offered as some kind of a “service.”

Hence, it can be seen that UA is gaining popularity in China, but urban residents' expectations have not been identified. A quantitative analysis of urban residents’ preferences is needed to sustainably develop UA. However, owing to the different penetration rates of UA in various regions of China at present, this study is limited to Shanghai.

Based on the above background, this study uses Shanghai as the study site to quantitatively evaluate Shanghai residents’ preferences for UA. A questionnaire survey was conducted among Shanghai residents in June 2022, with a final collection of 756 responses. Due to the coronavirus disease 2019 (COVID-19) pandemic, an online format was employed.

This study aims to develop UAs more successfully in Shanghai, specifically focusing on what kinds of UAs are preferred by residents, rather than preferences for specific UA facilities. This approach has three main benefits. First, by identifying residents’ preferences, a “win-win” situation can be achieved between residents and UA management. Second, for the government and municipalities, it allows them to better understand the position of UAs in urban development and define more flexible policies. This approach will lead to harmony between urban residents (consumers), UA management, and the government as well as the sustainable development of UA.

## 2 Methods

### 2.1 Geographical context

**Fig. 1:**
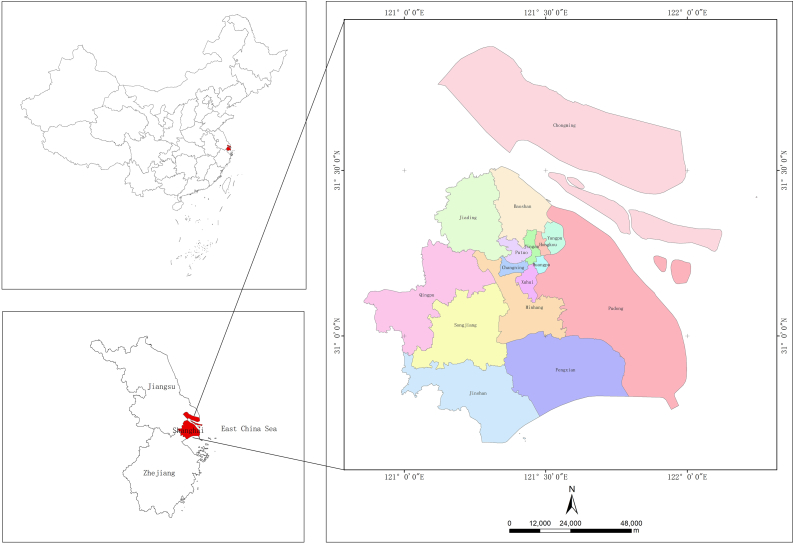
Map of the study region. (made by the authors).

As one of China's directly administered cities,^2^ Shanghai is located in the southeast coastal area and is connected to Jiangsu Province to the north and Zhejiang Province to the south (Fig. 1). Shanghai is the most developed city in China in terms of UA and has a high demand for UA owing to serious urbanization issues. As the most internationalized major city in China, Shanghai is also more flexible in its policies for new industries.

Various UA facilities have been operationalized in Shanghai. According to the results of a search on China's four major travel service websites (Ctrip, Tongcheng Travel, Fliggy, and Meituan^3^), UA facilities that are currently open to the general public in Shanghai can be broadly classified into three types based on management content and location: country parks, agri-tainments, and rooftop farms. As presented in Table 1, each of these has its own advantages and disadvantages. Country parks are usually large, serve as a food supply, and provide various recreational services. However, they are far from and have limited influence on the city center. The Jiabei Country Park in Jiading District, Shanghai, is a typical example of this type of UA. With a total area of 140,000 square meters, it is capable of large-scale agricultural production, but is more than an hour away from the city center. Agri-tainments in the suburbs were then developed by individual farmers around the old downtown areas. With the rapid expansion of urban areas, the remaining farmland was converted by private farmers into recreational farms that combine functions such as agricultural experiences and food accommodation. Although this type of UA is not as large as a country park, it is close to the city center and convenient in terms of transportation. The Lianyi Piba Ecological Park in Qingpu District is representative of this type of UA. The farm uses a green production system and allows urban residents to experience farming activities, such as loquat picking, and is equipped with restaurant and lodging facilities. The other type is the rooftop farm. This type of UA utilizes the rooftops of office buildings and condominiums in urban centers to manage mini-farms. This type of UA does not require additional land use and can improve the environment in urban core areas through plant cultivation. The Xiao Mi Feng Rooftop Farm and MYFARM Shimao Riviera in central Shanghai are typical examples. However, implementing this type of UA incurs a considerable cost. Currently, it is only implemented in some high-end apartment complexes and shopping malls; thus, the number of people who can benefit from this is limited.

**Table 1:** Classification and characteristics of UA in Shanghai.

	Country Park	Agri-tainment	Rooftop Farm
**Representative examples**	Jia Bei Country Park	Lianyi Piba Ecological Park	Xiao Mi Feng Rooftop Farm
			MYFARM Shimao Riviera
**Management** **Administrators**	Local government and private enterprise	Personal farmers	Private enterprise
**Location**	Remote suburbs	Suburbs or Urban-Rural Connection Zone	In town
**Original purpose**	Improvement of poverty issues	Agricultural land conservation	Vacant land utilization
	Provision of green space	Recreation	Recreation
**Advantages**	Very large in scale	There is a certain scale	Close to the city center
	Government-led, highly sustainable	Food supply available	Easy for residents to participate
	Food supply available	Environmentally friendly production is possible	All-organic production is possible
	Environmentally friendly organic production is possible	Relatively convenient traffic	
**Disadvantages**	Far from the city center	Degree of cooperation of individual farmers cannot be guaranteed	Small in scale
	Environmental effects are difficult to assess	Requires government support	High cost to implement
	Poor transportation	Environmental effects are difficult to assess	Low food supply function
			Limited number of participants

All existing UAs are either privately or corporately operated, and the provision of various services and participation fees are the responsibility of the management. Based on the publicly available price lists on the official websites of each UA facility and the amount of money spent in publicly available consumer reviews on major travel service websites, we calculated the average price of each type of UA service and summarized it in Table 2. However, because there are no clear requirements or indicators regarding market fixed prices for UA, the price differences between cases are quite large. Country parks and agri-tainments typically charge no entrance fees; instead, they charge fees for each service. The farming experience activity is generally the picking of crops. The price per person varies from 50 to 300 CNY depending on the type of crop harvested and the cultivation method.^4^ For instance, vegetables such as corn and sweet potatoes can be picked for 50 CNY; whereas, strawberries, loquats, and other fruits with high sales prices can cost approximately 300 CNY. Nonetheless, in all cases, farmers are allowed to take home the produce they pick. The prices of educational activities also vary depending on the content. For simple agricultural knowledge dissemination, participation is possible for less than 100 CNY, but for handmade activities, the price may be higher due to the cost of materials and other expenses. Conversely, rooftop farms usually charge admission fees. Depending on the service offered, the fee ranges from 200 to 400 CNY. Rooftop gardens in residential areas can charge monthly or annual fees.

**Table 2:** Fees for various UA services.

	Entrance Fee	Farming Experience	Educational Activities	Meal	Lodging	Total
**Country Park**	–	50–300 CNY	50–300 CNY	100–200 CNY	–	200–800 CNY
**Agri-tainment**	–	50–300 CNY	–	100–200 CNY	250–350 CNY	400–850 CNY
**Rooftop Farm**	200–400 CNY	–	–	–	–	200–400 CNY

*Note1: 1CNY≈0.14EUR (December 2022).

*Note2: The above information was obtained from the following websites searching keywords “country parks”, " agri-tainment " and “rooftop farm”. Ctrip official website: https://www.ctrip.com/; Tongcheng Travel official website: https://www.ly.com/; Fliggy official website: https://www.fliggy.com/; Meituan official website: https://www.meituan.com/.

*Note3: Some of the information is also referenced from the official websites of each UA facility. Official website of Jia Bei Country Park: #小程序://嘉北郊野公园/c2tmyofVsidBtGk; Official website of MYFARM Shimao Riviera: http://mp.weixin.qq.com/mp/homepage?__biz=MzA3Mjc3NjQ2Ng==&hid=1&sn=392f967418f7207fb9f496046422eb47&scene=18#wechat_redirect.

Most existing UA is implemented in commercial service facilities. According to the current land use classification standard,^5^ UA in China is divided into four categories: (1) agriculture in residential areas (RA), (2) agriculture in green spaces (GA), (3) agriculture in commercial and business facilities (BA), and (4) agriculture in other land use types. In particular, agriculture in residential areas is not adequately covered by the law, and agriculture in urban green areas is virtually non-existent in Shanghai. According to the above land use regulations, combined with our statistics on the search results of major travel websites, currently, the most common type of agriculture in Shanghai is commercial service facility land, to which the aforementioned country parks, agri-tainments, and rooftop farms belong.

### 2.2 Questionnaire design

As described in the previous section, we administered a questionnaire survey that included a series of choice experiments. Due to the survey's online format and guarantee of respondent anonymity, it was exempt from the requirement for ethical approval. We commissioned a questionnaire company to administer the online questionnaire. The questionnaire company first issued a simple screening questionnaire to a randomly selected portion of the 620 million registered members of the questionnaire site to confirm their age (20 years old or above), place of residence (Shanghai), gender and other information. At the same time, the screening questionnaire was used to select a group of respondents that matched the demographic composition of the city of Shanghai. During this period, the screened persons were not be informed of any information related to the study and the requirements of the respondents in this study. Screened participants received an invitation letter to participate in the study and those who agree to participate in the study received an official questionnaire. In total, the company sent invitations to 7254 people, and 765 people accepted the invitation and answered the questionnaire. More details are provided in Section 2.5.

Participants’ consent was obtained in written form. In the introductory section of the questionnaire, we explained to the participants the general contents and purpose of the questionnaire, and assured that all data collected would be used for academic research only. We ensured that the contents of the questionnaire only became available if the participant agreed to participate in the questionnaire.

Considering UA is composed of multiple attributes and the need to examine the value of each attribute, we used a method that accounts for multiple attributes at different attribute levels. For this purpose, we adopted the discrete choice experiment, which reflects the diversity of UA and is the most used method in the field of environmental economics. Compared to the contingent valuation method (CVM), another commonly used method for evaluating the value of non-market goods, choice experiments allow for the simultaneous evaluation of multiple attributes. Prior to the questionnaire, a pre-test was conducted among some residents to verify that the choice experiment was able to fulfill our research needs.

**Fig. 2:**
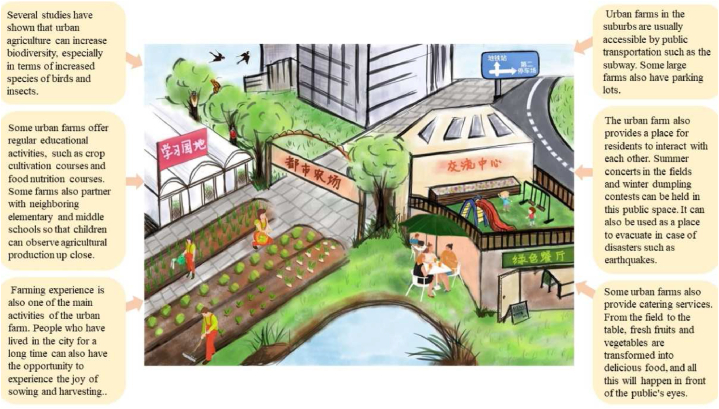
Explanation of UA and organic and green production (painted by the authors). (For interpretation of the references to color in this figure legend, the reader is referred to the Web version of this article.)

The questionnaire consists of four sections. In Section 1, we ascertained respondents' awareness of UA, and asked about their past participation and their evaluation of it. Thereafter, we briefly explained UA and organic and green production before moving on to Section 2 (Fig. 2). A series of designated choice experiments are performed in Section 2 to ascertain respondents' preferences. The third section, which appears to be extraneous to the core focus of this study, is dedicated to examining the preparedness for natural disasters. It aims to measure the extent of readiness among Shanghai residents and determine the variables influencing these preparedness levels. This investigation will draw upon quantitative and qualitative data sources to comprehensively understand disaster risk perceptions, preparedness behaviors, and the elements that influence them. These questions for another independent research study will not be used in this study. The content of the questions in this section is still visible in the full questionnaire. The last section recorded the respondents’ demographic characteristics. As mentioned in the previous section, the distribution and collection of questionnaires were conducted online^6^ and outsourced to a well-known Chinese online research firm (Wenjuanxing, Changsha Ranxing IT Ltd.).^7^ The survey was set up to allow respondents to respond using various devices, including smartphones, PCs, and tablets.

First, in Section 1, we ascertained how much respondents knew about UA. As UA is still not very popular in China, opinions about UA will likely differ between those who know nothing about it and those who know a lot about it. As past participation also influences preferences, we further asked experienced respondents what UAs they had participated in and how satisfied they were with their past experiences. As a survey of existing UA facilities in Shanghai revealed three main types of UAs (i.e., rooftop farms, agri-tainments, and country parks), we presented these three types of UAs as options in this study. Finally, respondents’ individual lifestyles also influenced their preferences. Hence, we also briefly asked about attitudes toward physical and mental health and the tendency to purchase environmentally friendly products.

**Fig. 3:**
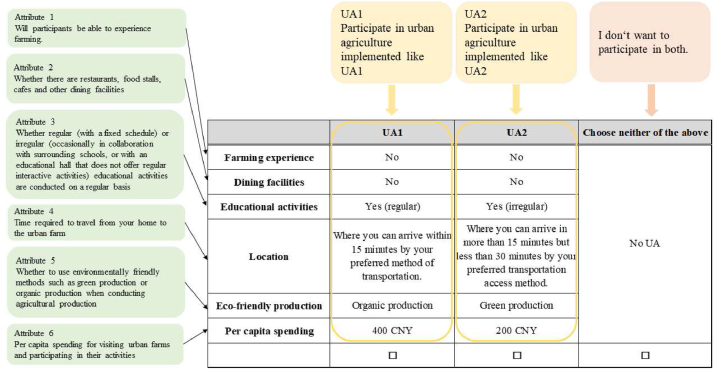
Example of a choice card.

The next section is Section 2, which includes a series of designated choice experiments and seeks to understand the respondents’ preferences. In each choice experiment, two UA initiatives composed of different attributes were presented, and respondents selected only one of the most desirable initiatives based on a balanced judgment of each item in each initiative. If both were undesirable, the respondent selected “Choose neither of the above.” Fig. 3 presents an example of the questionnaire utilized in the survey.

In the choice experiments, six attributes and two to four levels were established for each attribute as shown in Table 3. First, the attributes were set with reference to previous studies, and the six most important characteristics of existing UA facilities in Shanghai (provision of farming activities, dining facilities, educational activities, location, adoption of environmentally friendly production, and per capita spending) were considered. Initially, the provision of farming experience activities, establishment of dining facilities, and provision of educational activities were all set at two levels (Yes and No), but a pre-test was conducted to confirm the appropriateness of the settings. The findings showed that in terms of educational activities, consumers are more concerned with the way they are provided than with mere availability. Specifically, they examine whether these events are offered regularly, with a public schedule, or on an irregular basis (i.e., only on some holidays). Shanghai is a large city with diverse access to transportation. Many people have cars, but use public transportation to avoid traffic jams and parking problems. There are situations where people are far from a train station, but there is an expressway or other transportation nearby, so they will drive even if it takes 10 min to get there. Considering these situations, the location level was set to indicate the time required to reach the UA facility and use the transportation access preferred by the individual respondent. In addition to organic production, there is a production method called “green production” in China. The criteria for organic production are uniform worldwide, and it is a cultivation method that does not use chemical fertilizers, pesticides, or genetic modification technologies. Green production, according to the relevant provisions of “Environmental Quality of Origin of Green Food” issued by the Ministry of Agriculture and Rural Affairs of the PRC in 2021, can be briefly summarized as follows. Green production is based on the environmental friendliness and conservation of natural resources, and it allows the use of chemical fertilizers and pesticides with minimal impact on health and the environment. In terms of environmental friendliness, green production is better than ordinary production, but not as good as organic production. However, compared to organic production, green production is less expensive and technically easier to perform and is currently quite popular in China. Therefore, green production is one level of production in addition to organic production. Then, per capita spending was averaged based on what was published on the official website of each UA facility and each review site.

**Table 3:** Attributes and attribute levels for the choice experiment.

Attributes	Level
Providing hands-on cultivation activities	Yes; No
Establishment of dining facilities	Yes; No
Provision of educational activities	Regular offerings; Irregular offerings; None
Location	Where you can arrive within 15 min by your preferred method of transportation.
	Where you can arrive in more than 15 min but less than 30 min by your preferred transportation access method.
	Where it takes more than 30 min to reach your destination by your preferred transportation method.
Adoption of eco-friendly production	Adopt organic production; Adopt green production; None
Per capita spending	100 CNY; 200 CNY; 400 CNY; 850 CNY

When the orthogonal design was conducted under the above settings, there were 72 results, and because the number of questions would be quite large if all of them were used, 24 were randomly drawn from the 72 combinations and divided into four sets to avoid burdening the respondents too much. Thus, there were four versions of this questionnaire, each containing six choice experimental questions.

The last section presented the respondents’ demographic characteristics. In addition to basic attributes, such as gender, age, and monthly household income, the respondents were asked whether they had children (preschool, primary, and secondary school students, and high school students) and whether they had worked in agriculture-related jobs. The collected data were compared with the census data to verify whether the sample adequately represented the population of the study area. The details are outlined in Section 2.5.

### 2.3 Empirical modeling

To estimate residents’ preferences for UA in Shanghai, we utilized the conditional logit (CL) and mixed logit (ML) models. The CL model is a more basic model, wherein the homogeneity of preferences among individuals is assumed [22], which is usually not the case in reality. Moreover, the CL model assumes the independence of irrelevant alternatives (IIA), a very strong assumption that is often violated. If the IIA assumption does not hold, the estimates from the CL model are biased and invalid. It is widely known that if the IIA assumption does not hold, estimates from the CL model will be biased. An alternative approach, the ML model, eliminates this problem by allowing for random preference changes, unlimited alternative patterns, and correlations between unobserved factors over time [23].

In the ML model, the utility of $U_{ij}$ when resident i chooses UA j is expressed as follows:

(1) $$U_{ij}=V_{ij}\left(\beta_{i}\right)+\epsilon_{ij}$$

In Equation (1), $\beta_{i}$ represents the utility parameter for resident i. Assuming that $\epsilon_{ij}$ follows an independent and identical Gumbel distribution, the probability $L_{ij}\left(\beta_{i}\right)$ that resident i with utility parameter $\beta_{i}$ chooses UA j can be expressed as follows:

(2) $$L_{\text{ij}}\left(\beta_{i}\right)=\frac{\exp\;\left(V_{\text{ij}}\left(\beta_{i}\right)\right)}{\sum_{k\in C}\;\exp\;\left(V_{\text{ik}}\left(\beta_{i}\right)\right)}$$

Notably, the utility parameter $\beta_{i}$ of each individual is unobservable. Hence, the integral of the CL model is considered for the density of the utility parameter $\beta_{i}$. The probability of resident i to choose UA j is expressed as follows:

(3) $$P_{ij}\left(\Omega \right)=\int L_{ij}\left(\beta_{i}\right)\cdot f\left(\beta |\Omega \right)d\beta$$

where f(β|Ω) represents the probability density function of β, and Ω denotes a vector of parameters that characterize this probability density function. Because this equation is not a closed form, the optimal parameters cannot be solved numerically. We employ the simulated maximum likelihood for finding the optimal parameters of the equation. Stata 18 and add-in package ‘mixlogit’ were used.

The parameters estimated from Equation (3) can be used for conducting policy simulations. More specifically, the estimated parameters can be plugged into logit formula (Equation (2)) to obtain respondents’ choice probabilities for UAs with different conditions. Using this method, we set up four UAs with different conditions and predicted how the choice probabilities for each UA would change at the payment level. This simulation method can be easily implemented using mixed logit results and provides useful information for discussing desirable UA for society. See Section 4.2 for details.

### 2.4 Explanatory variables

Based on the attribute and level settings in the choice experiments, nine UA attribute variables were established, and all eight, except Pay, were dummy variables. Farming and Food indicate whether farming experience activities are provided and whether dining facilities are available or not, respectively, with 1 for “Yes” and 0 for “No.” EduR indicates regular educational activities and is 1 when there is a published timetable or schedule and educational activities are held regularly and 0 when they are not regular or do not hold educational activities. Contrariwise, EduI signifies the provision of irregular educational activities and is 1 only when irregular educational activities are held. Dis15 and Dis30 are two variables that indicate the location. Using “where you can arrive within 15 min by your preferred method of transportation” as the baseline, Dis15 and Dis30 indicate “where you can arrive in more than 15 min but less than 30 min by your preferred transportation access method” and “where it takes more than 30 min to reach your destination by your preferred transportation method,” respectively. EnG and EnO reflect the status of the adoption of green and organic production. If they were adopted, they were set to 1; otherwise, they were set to 0. Finally, Pay represents per capita participation spending.

In addition to the above variables, we also set four types of individual attributes and a reciprocal variable, with the alternative specific constant (ASC) as the control parameter. Each of these four attributes reflects whether the respondent is highly educated (ASC × Edu), has children under the age of five years (ASC × Child5), has children between the ages of six and 15 years (ASC × Child15), and has agricultural work experience (ASC × WExp), all of which are dummy variables. The corresponding variable value is 1 if the respondent has the above attributes, and 0 otherwise.

### 2.5 Survey overview and sample demographics

We outsourced our survey to Wenjuanxing, a well-established survey firm in China. From their more than 6.2 million registered members of the questionnaire site in the country, 7254 adults residing in Shanghai were selected by considering the age structure of the population. Of them, 756 valid responses were obtained. Although the valid response rate (10.4 %) is not high, given the peculiarities of the implementation of online questionnaires, their response rates are usually not as good as those of offline questionnaires [[24], [25], [26], [27], [28]], we consider the response rate for this survey to be reasonable. The number of responses received for each version was 188, 189, 188, and 191, respectively.

Table 4 compares the sample with the population of the study area. Key attributes included sex, age, education, and monthly income per household. Among these, the proportion of the population in their 30s and the proportion of the population with university and higher education are slightly higher. As this survey was conducted in an online format, this result is reasonable considering that the Internet user base is composed of people in their 30s and many of them have higher education. In terms of other attributes, the sample utilized in this study reasonably reflected the characteristics of Shanghai.

**Table 4:** Characteristics of our sample and study region.

	Sample	Study Region
% of the female population	50.53 %	48.20 %
% of people aged 20–29 years	15.74 %	17.12 %
% of people aged 30–39 years	32.41 %	23.19 %
% of people aged 40–49 years	14.29 %	16.73 %
% of people aged >50 years	37.57 %	42.96 %
% of college graduates and higher	55.16 %	33.87 %
Average monthly income per household (CNY)	17,503.97	13,964.85

*Note: Data for the study region were taken from the “Shanghai Statistical Yearbook 2021.”

## 3 Results

### 3.1 Estimated results

Table 5 summarizes the results of estimating respondents’ choice behavior with the CL and ML models based on the data obtained in the choice-type experiment; the CL and ML1 models include only attributes and ASC for UA, while the ML2 model includes as control factors four types of individual attributes and a reciprocal variable, with the ASC as the control parameter. Comparing the above three models, the AIC of ML2 is the lowest, and many of the added variables are also significant; therefore, we report the results based on this model.

**Table 5:** Analysis results for conditional logit model and mixed logit model.

Dependent Variable		Conditional Logit (CL)		Mixed Logit 1 (ML1)		Mixed Logit 2 (ML2)	
CHOICE		Coefficient	Std. Error	Coefficient	Std. Error	Coefficient	Std. Error
Mean	ASC	−0.959***	0.101	−2.355***	0.183	−1.459***	0.201
parameters	ASC × Edu	–	–	–	–	−0.769***	0.154
	ASC × Child5	–	–	–	–	−0.428	0.284
	ASC × Child15	–	–	–	–	−0.536	0.216
	ASC × WExp	–	–	–	–	−1.060**	0.369
	Farming	0.360***	0.058	0.525***	0.074	0.528***	0.075
	Food	0.346***	0.052	0.495***	0.061	0.496***	0.062
	EduR	0.501***	0.064	0.637***	0.078	0.639***	0.078
	EduI	0.171**	0.065	0.308***	0.076	0.310***	0.077
	Dis15	−0.138*	0.055	−0.143*	0.063	−0.144*	0.065
	Dis30	−0.334***	0.059	−0.429***	0.066	−0.430***	0.066
	EnG	0.861***	0.053	1.019***	0.063	1.021***	0.063
	EnO	0.838***	0.063	1.053***	0.075	1.057***	0.077
	Pay	−0.002***	0.000	−0.002***	0.000	−0.002***	0.000
S.D.	ASC	–	–	2.589***	0.155	2.463***	0.184
parameters	ASC × Edu	–	–	–	–	0.088	1.706
	ASC × Child5	–	–	–	–	−0.457	1.315
	ASC × Child15	–	–	–	–	0.135	2.276
	ASC × WExp	–	–	–	–	0.941	0.911
	Farming	–	–	−0.618***	0.156	0.633***	0.156
	Food	–	–	−0.016	1.717	−0.041	1.042
	EduR	–	–	0.480*	0.211	0.481*	0.213
	EduI	–	–	−0.064	1.078	−0.073	0.957
	Dis15	–	–	0.036	1.347	0.166	0.485
	Dis30	–	–	0.020	1.413	0.019	1.304
	EnG	–	–	−0.072	0.643	0.002	1.158
	EnO	–	–	0.423**	0.150	0.414**	0.153
							
	# of obs.	13608		13608		13608	
	# of cases	4536		4536		4536	
	Log-Likelihood	−4164		−3831		−3819	
	AIC	8347		7701		7691	

*Note: *, **, and *** indicate statistical significance at 10 %, 5 %, and 1 % percent, respectively.

In the mean value parameter of the ML2 model, all variables are statistically significant except for one interaction term with the ASC. Since the ASC (−1.459) is the choice to not support any of the presented UAs, its negative and significant value indicates that overall respondents support the UAs and tend to choose one of the presented UA scenarios, relaying that respondents tend to choose one of the scenarios. Pay, the amount paid, is also negative and significant, denoting that the utility gained via UA decreases as the amount increases.

All attributes related to the UA scenario were dummy variables and all their mean value parameters were significant. Comparing the individual values, the coefficient values for EnO (1.057) and EnG (1.021) are particularly high, followed by EduR (0.639), farming (0.528), food (0.496), and EduI (0.310). The negative parameter with the highest value is Dis30 (−0.430), followed by Dis15 (−0.144). However, the significance of Dis15 was at the 10 % level and should be interpreted with caution. This point is addressed in Section 4.1.

Among the four individual attribute variables utilized as cross terms in the ASC, higher education (−0.769), children between the ages of six and 15 years (−0.536), and agricultural work experience (−1.060) were negatively significant; the variable representing the attribute of having children under the age of five years was not significant.

The coefficients of the standard deviation parameters (S.D. parameters) indicate valuations of resident preferences. When the S.D. parameter of a variable is statistically significant, it indicates that there is variability in resident preferences regarding this variable. The S.D. parameter of ASC (2.463) is positively significant and has a high coefficient value, which means that there is variability in the public's preference for UA. In other words, different people have different preferences. The S.D. parameters for farming experience activities (0.633), regular educational activities (0.481), and organic production (0.414) were significant. As the S.D. parameters for the other UA attributes are not statistically significant, we can say that the homogeneity of resident preferences is high for many UA attributes. Otherwise, the ASC crossover term displaying individual attributes were not significant, which means that residents with different personal attributes have consistent preferences for UA.

### 3.2 Marginal willingness to pay (MWTP)

Table 6 outlines the MWTP for each UA attribute, and the results are all statistically significant: except for ASC, residents’ MWTP for green production and organic production is the highest, at 454.45 CNY and 442.34 CNY, respectively, followed by regular educational activities (264.60 CNY), farming experience activities (190.01 CNY), and dining facilities (182.63 CNY). The MWTP for non-routine educational activities also had a positive value (90.45 CNY) but was much lower than that for regular educational activities. The MWTP for locations that can be reached within 15–30 min and those that take longer than 30 min are −72.66 CNY and −176.54 CNY, respectively.

**Table 6:** MWTP for each UA attribute.

	MWTP
ASC	−506.53***
Farming	190.01***
Food	182.63***
EduR	264.60***
EduI	90.45**
Dis15	−72.66*
Dis30	−176.54***
EnG	454.45***
EnO	442.34***
scalePar	0.002***

*Note: *, **, and *** indicate statistical significance at 10 %, 5 %, and 1 % percent, respectively.

Our estimates were higher than those reported in previous studies. For example, Gustavsen et al. (2021) showed that more than half of the residents were willing to pay 50–500 NOK (about 4.7–47 EUR) per year for UA in Oslo, Norway [8].^8^ In a survey of Seoul residents [29], the total MWTP of UA was approximately 18,852 KRW (approximately 13.8 EUR). Sanyé-Mengual et al. (2018) found that the WTP for UA products for all residents is approximately 78 % of that for general products, and the willingness to buy UA products is about the same as for general products [18]. A survey in Berlin, Germany has similar results [30]. The above shows that the value of the MWTP estimated in this study is somewhat higher than that in previous studies. This result can be interpreted from three perspectives: the level of consumption in Shanghai, the leisure facility character of UA, and the influence of COVID-19, which is consistent with the actual situation in Shanghai. This is discussed in detail in Section 4.1.

## 4 Discussion and policy simulation

### 4.1 Discussion

The results of the above analysis provide several insights into the general public's preference for UA in Shanghai, China. The coefficients and significance of the mean parameters outline that ASC is negative and significant; therefore, the respondents overall support UA and tend to choose one of the UA scenarios presented as discussed in Section 3.1. This finding reflects the general public's positive preference for UA, and is consistent with the results of previous studies conducted in Japan, Korea, and Italy [17,18]. We also considered the following reasons for these results. Shanghai has the highest degree of internationalization in mainland China, and its residents are inherently more receptive to new things. Furthermore, residents' environmental awareness is high [31]. Especially in recent years, the environmentally friendly lifestyle represented by low carbon living has been promoted globally, and many people in Shanghai are committed to living this lifestyle [32]. UA has been garnering attention in Asian countries in recent years and is a representative example of such a lifestyle in practice. It can be said that the UA is a representative example of this lifestyle.

Among the attributes related to UA, the coefficient values were particularly high for organic (1.057) and green production (1.021). This highlights a preference for UAs that adopt environmentally friendly production rather than conventional agriculture, which emphasizes efficiency. The two reasons for this are food safety and environmental needs in urban areas. Urban residents generally value food safety. In a study of residents of Taegu, Korea [17], “safe food production” was frequently indicated among the motivations for residents to participate in UA. Luehr et al. (2020) also noted that food safety assurance was the main motivation for Nanjing residents to do UA [15]. In China, while the consumption level of urban residents has increased, frequent food safety issues (e.g., the melamine milk contamination incident in 2009) have made consumers pay increasingly more attention to food safety issues [33,34]. At the same time, numerous studies over the years have shown that pesticide residues in food are a major cause of food safety problems, and that organic production or production using fewer chemical fertilizers, pesticide and herbicides can significantly improve food safety [[35], [36], [37]]. Therefore, when it is an established fact that urban environments are more complex than rural ones, urban residents are justified in choosing environmentally friendly production that uses no or fewer chemical products for greater food safety. It is also assumed that UAs are “located in urban areas.” Previous studies show that the most common reason for opposition to UA and UA products is pollution from agricultural production [18,30,[38], [39], [40]]. In addition to the pollution from fertilizers, pesticides and herbicides just mentioned, agricultural production also inevitably generates sewage and waste. Including effluent discharges due to irrigation and various types of organic wastes generated during the cultivation process. As urban areas are usually densely structured, pollution from agricultural production in the immediate area can affect the surrounding residents more quickly and directly. While having a nearby UA makes it convenient to participate in a variety of activities, some residents may be concerned about whether the use of chemical fertilizers in agricultural production will pollute the water, air, and soil in the surrounding area. Shanghai is a large city with a population of over 24 million and a high residential density in the central area. If one is going to farm in such a place, environmental considerations of the surrounding areas are crucial.

The estimated results highlight a small difference in the coefficient values between organic and green production. In reality, the penetration of green and organic products remains low in China. According to data published by the National Bureau of Statistics,^9^ the total sown area of crops in China in 2021 is expected to be 168,700,000 ha. The area under organic crops was 2.756 million hectares, accounting for approximately 1.5 % of the total sown area [41]. Contrarily, the average penetration rate of organic farming in Western countries is approximately 2–5%; whereas, the environmentally captured area of green production is 5,758,000 ha, or approximately 3.4 % of the total sown area [42]. As previously discussed, while both green and organic production are environmentally friendly, they are not judged based on the same criteria, with organic production being completely free of chemical pesticides, insecticides and herbicides, while green production allows for the use of some of products that have a lesser impact on the environment. Due to the relatively low popularity of both types of production in China, the market rate of the products is also relatively low, and the publicity information received by most ordinary consumers is also very limited. According to the results of the questionnaire survey we implemented, more than half of the respondents indicated that while they were aware of green and organic production, they were not familiar with the specifics. Hence, for the majority of the public, low awareness due to lack of information makes it difficult for them to differentiate between green and organic production. In other words, when making a choice, they would prefer environmentally friendly production, but would not specifically prefer one or the other.

UA facilities that offer farming experience activities and complete dining are more desirable. As mentioned in the Introduction, UA facilities in China are considered leisure facilities in the majority of cases. The purpose of residents’ participation in UA is to spend their leisure time. The more services a UA facility offers, the more it is preferred by the residents. Farming experiences activities can give people a better sense of how to grow crops in urban areas. The establishment of dining facilities will provide more leisure space for participants and deepen the image of local production for local consumption.

Residents prefer UAs that hold regular educational activities over irregular educational activities. In general, residents prefer the educational function of UA [17,18]. As UA facilities with educational functions are more readily accepted by residents, educational activities can ensure resident participation rates [30]. At the same time, residents want to be able to actively choose when to participate in educational activities. Regularly scheduled educational activities, however, usually have a publicly available schedule so that residents know exactly when and how often the activities will be held to make it easier for them to choose the right time to participate. Irregularly organized activities, on the other hand, are very passive in terms of scheduling, as residents have no guarantee that they will be available when the activity is held, or that the activity will be held when they are available. As a result, although educational programs are generally popular, UA facilities that host educational programs on a regular basis are preferred by residents.

Regarding location, overall, the closer the UA facility, the greater the preference, but it does not have to be right in front of it. Dis30 is significant at the 1 % level, and when it takes more than 30 min to get to a UA facility, residents' willingness to participate decreases. In contrast, Dis15 is significant at the 10 % level, meaning that when the time it takes to get to a UA facility is between 15 and 30 min, residents' utility may not decrease. In other words, when a UA facility can be reached within 30 min, the change in the distance has a smaller impact on residents’ preferences. Therefore, 30 min is a “watershed” for the location of UA facilities.

In conjunction with the survey results, the UA participation rate was not very high, but the residents were quite satisfied with the UA facilities they had visited in the past. About 40 % of respondents reported that they had participated in UA in 2021. A 40 % participation rate may be high for UA in other countries, but in China, UA is usually viewed as a recreational facility, different from the UA that is generally prevalent in other countries. So, we believe that a 40 % or even higher participation rate is reasonable. According to the China Urban Modern Agriculture 2021 released by Shanghai Jiao Tong University^10^, the city with the highest modern urban agriculture development index in China is Shanghai [43]. UA that combines leisure and recreation functions and uses modernized production technologies has developed particularly well in Shanghai. In this context, considering the attributes of UA as a recreational facility in China, and the actual situation in Shanghai (high income, high consumption level, and high recreational demand level), the participation rate of the public should be higher. Meanwhile, a comparison of the number of reviews on major travel sites shows that the number of consumer reviews have decreased considerably after 2020. Thus, although the UA participation rate was somewhat lower than expected, the impact of COVID-19 is considered an important cause. Residents participated the most in agri-tainment. Satisfaction with UA was high, with 98.7 % of respondents being satisfied with the UA facilities they had visited in the past. These individuals have more positive attitudes toward UA and are more willing to participate in the future. This finding is consistent with the results of a previous study [17].

Those in the higher education group, those with small- and medium-sized students, and those with agriculture-related work experience have stronger preferences for the UA. The results of the ASC cross-term analysis show that among the four individual attributes, work experience has the strongest effect on preferences. Those who have worked in agriculture in the past have a better understanding of UA and are more likely to participate as experts. Education had the next strongest influence. Educated people are usually more receptive to new things and more concerned about social issues, including environmental issues. They have a greater interest in UA and UA products [30]. Regarding the presence of children, those with children between the ages of six and 15 years are more likely to participate in UA, while those with children under the age of five years are less likely to do so. Elementary and middle school students can experience agricultural activities and acquire agricultural knowledge at the same time by participating in UA. In contrast, it is difficult for children under the age of five years to make substantial use of the various functions of UA. Thus, while those with elementary and middle school students might participate in UA for the sake of their children, those with young children are less likely to do so.

Based on the coefficients of the S.D. parameters, we can easily see that regarding UA attributes, there are some UA attributes with varying resident preferences, but from an overall perspective, there is a high homogeneity of resident preferences for many UA attributes. Farming and EnO were significant at the 1 % level. Some residents have strong preferences for farming experience activities and organic production, while others are less desirous. For those who have previously worked in agriculture, farming experience activities may not be anything special, but for those who have not, this may be new and interesting. Moreover, Edu is also significant but at the 10 % level of significance. Given that these educational activities are usually geared toward children, residents with children have a strong preference for regular educational activities, whereas those without children do not. While most of the S.D. parameters of UA attribute variables are insignificant, which suggests that residents have similar preferences for the vast majority of UA attributes. This indicates a general preference for the implementation of urban agriculture in the neighborhood and a preference for urban agriculture facilities that offer a wide range of services and use environmentally friendly production methods. Nevertheless, the ML model produces a more valid result than the CL model in this study,^11^ indicating the existence of heterogeneity.

Combining both the ASC variable and the UA attribute variable in the S.D. parameter coefficients, it shows that residents have different emphases when discussing UA preferences. In other words, some residents are more concerned about the provision of dining facilities in UA facilities than the provision of educational activities. There are also residents who prioritize the provision of educational activities.

However, the provision of farming experience activities and organic production are the two attributes that have received the most attention in previous studies and are also points of interest in this study. Therefore, to comprehensively explore the specific impact of both of these on UA, we conducted a policy simulation. The details are presented in Section 4.2.

Finally, the estimated MWTP values were somewhat higher than those reported in the previous studies. This outcome can be attributed to three reasons.

First, UAs in China have leisure facilities, and visitors spend a considerable amount of money. As mentioned in the Introduction, UA in the West emphasizes the cultivating behavior of urban residents. Conversely, UA in China emphasizes recreational functions, and people usually go only on weekends and holidays. Hence, many residents attend “only a few times a year,” and their expected consumption per visit to UA is also high. For comparison, the admission fee to Shanghai Disneyland is approximately 435–545 CNY per visit,^12^ and the per capita spending is approximately 700–900 CNY when food, beverages, and other spending inside the park are added.^13^ Thus, if we consider UA as a type of amusement park, the MWTP estimates in this study are equivalent to the per capita consumption of Shanghai Disneyland. Table 2 also presents that the value of each UA service in Shanghai is, on average, high, although it varies from facility to facility.

Second, as a major city in China, residents of Shanghai spend more time there. In terms of GRP (gross regional product), Shanghai ranks first in China, according to the National Bureau of Statistics. According to the Shanghai Statistical Yearbook (2021), the average monthly income of a household in Shanghai is 13,964.85 CNY [44]. Although the estimated MWTP value is slightly high, we believe that there is not much deviation compared to the economic and consumption levels of Shanghai itself.

Third, residents prefer local recreational facilities because of the behavioral restrictions imposed by COVID-19. Tourists tend to avoid long-distance travel, emphasizing the risk of infection at their destination [45]. A study conducted in Hong Kong noted a significant increase in local travel and recreational activities [46]. Since the start of the pandemic, China has adopted stringent measures. Specifically, a nationwide system of “Health Codes,”^14^ which reflect individual behavior, has been utilized and many restrictions on personal outings have been placed on individuals. This stringency has led to a tendency for residents to use local recreational facilities, as going outside their hometowns may cause inconvenience in their daily lives as the health code reflects a higher risk of infection for the individual. The survey results suggest that many residents expect UA effects during the pandemic; 73.54 % of respondents agree that UA can provide food supply during a pandemic, and 65.61 % agree that UA can be enjoyed as a recreational activity during quarantine. The shortage of food supplies during the COVID-19 pandemic is a well-recognized fact, especially in urban areas where strict out-of-home restrictions were generally enforced, and the desire of residents to have access to fresh food close to home had intensified, leading to heightened expectations for UA among those who have experienced COVID-19 [47]. Similarly, opportunities for people to go outside during a pandemic are significantly reduced. In China, most urban residents live in apartments, which means that there are no separate courtyards or private outdoor spaces to move around in. Neighborhoods with UAs provide at least a limited amount of outdoor space for people in the community. As a result, after months of being banned from going outside, urban dwellers generally want outdoor space in their neighborhoods.

Notably, the COVID-19 pandemic did have an unavoidable impact on the behavior of Shanghai residents, but this does not mean that the results of this study are seriously biased. Since the global outbreak of the COVID-19 pandemic in 2020, the world has been affected to varying degrees and, objectively, the impact of the pandemic has been far-reaching and unavoidable as it has lasted over a long period of time. The COVID-19 pandemic impacted all aspects of people's lives, changing their behavior patterns [48]. In terms of leisure activities, people tend to avoid long-distance travel and prefer local recreational activities [45,46]. To prevent the spread of infection in China, many people avoid going out, and leisure activities have decreased significantly [49]. These effects are not exclusively short-term, but are likely to be long-term. Although strict COVID-19 rules are no longer in place globally, many people continue the habits they had during the pandemic, such as working from home and shopping online. Thus, the high expectations for UA due to COVID-19 do not diminish with the end of the pandemic. The results of this study appropriately reflect attitudes toward UA after experiencing the COVID-19 pandemic.

### 4.2 Policy simulation

Based on the results of the ML2 model, policy simulations were conducted to calculate residents’ acceptance of UA under different conditions. Specifically, we calculated the average probability of a resident choosing UA based on the results of the mean parameters. The following four conditions were considered: 1) farming experience activities + organic production; (2) no farming experience activity + adoption of organic production; 3) farming experience activities + conventional production; and 4) no farming experience activity + conventional production. For each scenario, we predict how the acceptance probability changes for different payment levels.

**Fig. 4:**
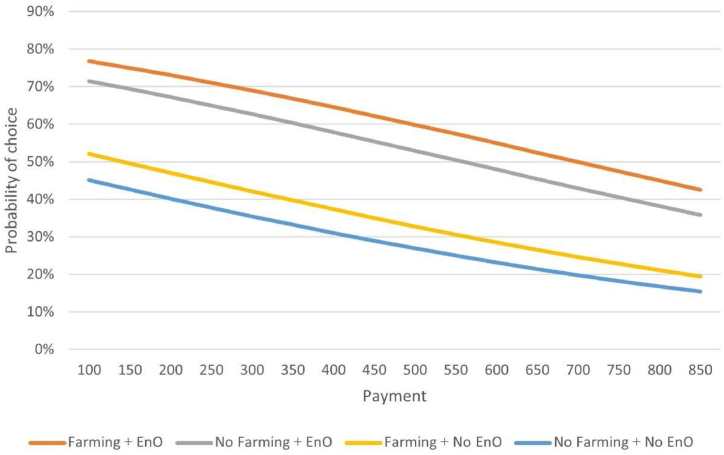
Simulated adoption rates of UA for different result requirements based on ML2 results.

The results are shown in Fig. 4. The horizontal axis represents the price, and the vertical axis represents the probability of UA being selected. This figure illustrates that UA becomes less accepted when payments increase, but the level of acceptance is higher with farming experience activities and organic production. In the case of farming experience activities + organic production adoption (Condition 1), the average acceptance level of UA was approximately 60 %. Contrariwise, the average level of UA acceptance is approximately 29 % in the case of no farming experience activity and non-adoption of organic production. The average probabilities of UA being selected under Conditions (2) and (3) are 54 % and 35 %, respectively. In other words, the development of farming experience activities and adoption of organic production can improve the UA acceptance level, and the influence of organic production was particularly strong.

When viewed together with the estimated MWTP, the MWTP is approximately 632.44 CNY (190.01 CNY + 442.34 CNY) for the case of farming experience activities + adopting organic production (Condition 1), which is about 55 % UA acceptance. In the case of no farming experience activity + organic production adoption (Condition 2) and farming experience activities + organic production non-adoption (Condition 3), the MWTP is 442.34 CNY and 190.01 CNY, respectively, and the UA's acceptance level is approximately 54 % and 45 %. In other words, the UA acceptance rate is approximately half when providing farming experience activities or adopting organic production.

As shown in Table 2, the average UA fee in Shanghai was approximately 500 CNY. Although the services offered and pricing varies from one UA facility to another, the prices tallied in Table 2 are a relatively objective figure when combined with the per capita spending publicly available on major travel websites. Therefore, we choose the average of the prices shown in Table 2 to represent the average payment of the public on UA participation. When payment is 500 CNY, the acceptance level of UA with farming experience activities and organic production adoption was approximately 60 %. Under Conditions (2) and (3), the UA acceptance rates were 53 % and 33 %, respectively. UA facilities with no farming experience activity and no adoption of organic production had an acceptance rate of only 27 % under the same price. In other words, the adoption of organic production can double UA's acceptance level.

## 5 Conclusion

This study examined Shanghai residents' preferences for UA, quantitatively analyzed each UA attribute, and calculated the MWTP for each attribute. The effects of each UA attribute on residents’ adoption probability and the diversity of resident preferences were also examined. UA is still in its infancy in Shanghai but has a high development potential. This study makes the following recommendations for the sustainable development of UA.

First, UA management should provide more UA services. Residents tended to choose UAs that offered diverse services. Additionally, UA in China has the characteristics of leisure facilities, and when residents participate in UA, they often desire to enjoy various UA services. The more services offered while running a UA, the more residents are attracted. In particular, residents’ preferences for farming experience activities are not consistent, but the results of the policy simulation show that the provision of farming experience activities has a positive impact on the acceptance of UA. Shanghai is well known for its economic development. According to the questionnaire, 30.7 % of respondents had work experience in agriculture, and 78.6 % had farming experience as a hobby. It can be assumed that these individuals are more likely to participate in UA if they have farming experience. Offering farming activities can effectively attract residents to participate in the UA. Given that the emphasis on UA attributes varies from resident to resident, communication strategies need to be noted when promoting UA. UA management should implement appropriate communication strategies for consumers with specific preferences based on the content of their own service offerings.

As residents show a strong preference for environmentally friendly production, it is advisable to give full consideration to the environment and prioritize green and organic production when conducting agricultural production in the UA. As mentioned above, Shanghai, which has become China's main city, is undergoing rapid urbanization and the resulting environmental problems are serious. In particular, environmental improvement in city centers, where the population is concentrated, has been a priority for residents in recent years. More than 80 % of the respondents reported that UA could improve biodiversity and air quality. Hence, in addition to being a recreational facility, the environmental improvement effects of UA on urban areas are highly anticipated. The findings of the policy simulation also show that residents tend to visit environmentally friendly production.

The government must provide appropriate financial support to UA operators, especially those who adopt green and organic production. While UA facilities that adopt environmentally friendly production are welcomed by the public and are beneficial to the improvement of the urban environment, they may also face high management risks on the part of operators. Green or organic production typically incurs higher costs than general agricultural production. In particular, organic production is strictly prohibited from using chemical fertilizers, which, in turn, requires considerably more labor than general farming [50]. Besides, the agricultural externalities (environmental considerations) of adopting environmentally friendly production are usually not reflected in the price of the product [51]. If management simply imposes high costs on the participation fee (on the consumer side), then consumers may choose not to participate. Government support is vital for avoiding this situation. Currently, policies related to organic farming in China are primarily formulated and implemented by local governments, and specific guidelines at the national level are lacking. Although many policies are supporting organic farming in various provinces, these are mostly limited to reduced certification fees and subsidies for production materials due to imprecise and inadequate decision-making rationale [52]. Strong financial support is needed to encourage UA operators to adopt proactive, eco-friendly production practices.

However, more flexible and clear-cut measures in terms of land-use regulations are expected. Although organic production and other environmentally friendly production methods are usually difficult to scale up, large-scale production is not essential for UA itself. From this perspective, organic production should be easily promoted in UA. However, in reality, in addition to the cost factors mentioned above, there are also restrictions on land use regulations in China that hinder the adoption of organic production. The cycle of cultivation emphasized in organic production often conflicts with local land-use regulations [50]. Furthermore, this problem often occurs not only in the promotion of organic production but also in the implementation of UA itself [15,21]. Shanghai is a flexible city in terms of various policies and has provided policy support for the promotion of in many aspects in recent years. The Shanghai government's support is mainly in the form of land use permits, issued by the local government, for which the criteria are generally more lenient. The Shanghai government encourages the flexible use of urban space to operate UA. Furthermore, the Action Program for Promoting High-Quality Agricultural Development in Shanghai (2021–2025) was issued in 2020, in which district and county units were asked to adequately safeguard the demand for UA land.^15^ However, since urban agriculture in China is still in its infancy, many relevant variables, such as land use policies, still need to be improved [53]. It is not very clear in the national land use policy which land can be used for UA operations and which cannot, which limits the growth of UA. If the local government adopts a relatively conservative attitude, UA operations can be difficult to develop due to land use constraints. Therefore, in order for UA to continue to develop and grow, the section relating to UA land usage in the national land policy must be improved.

Additionally, environmental regulations regarding UA must be perfected. Failure to complete UA control measures could lead to further environmental pollution [54]. This issue was not addressed in this study, but has been mentioned in many previous studies. Overall, UA implementation is beneficial for improving the urban environment; however, as mentioned earlier, some residents do not accept UA because of pollution considerations [18,30,[38], [39], [40]]. As UA is intended to be implemented in urban areas, attention must be given to environmental needs and restrictions in urban areas. It is critical to consider the opinions of the inhabitants of an area when implementing UA in the vicinity of residential areas. Although this factor is not the primary determinant affecting the implementation of UA, a well-developed environmental policy is imperative for its sustainable development.

Before concluding this paper, we point out the limitation that was not addressed in this study. This study is geographically limited because it covers only the city of Shanghai. Although UA has been relatively well-developed in Shanghai, given the real situation of large regional variation in China as a whole, the results of this analysis are not likely to be applicable in other regions. As a continuation, the conclusions of this study should be validated in other regions.

At the same time, we were not able to conduct further comparative studies between Shanghai and other regions because this study only obtained data about Shanghai and did not conduct the same questionnaire survey in other regions. However, we believe that it would be interesting to compare the motivation of Shanghai citizens to participate in urban agriculture with that of other regions in China and even globally (e.g., Africa). This will be one of the directions for future research.

Another limitation of this study is related to the respondents' experience in agriculture. Thirty-one percent of the respondents in this study indicated that they have experience in agriculture-related practices, and 80 % of the respondents said that they do gardening or farming in their leisure time out of interest. As we described in Section 2.5, the sample used in this study fits the demographic characteristics of Shanghai. Based on this, we infer that the population of Shanghai residents with agricultural experience is about one-third of the city's population. However, this is not included in the available official public data, so we cannot confirm whether the extrapolation is entirely correct. If the proportion of the population with agricultural experience is less than 30 % in reality, then in reality the public's expectation of UA may be slightly lower than the extrapolated results of this study. Conversely, the public's expectations may be higher.

Once again, we would also like to emphasize that COVID-19 has had an unavoidable impact on this study. This does not mean that the results of this study are unrepresentative; on the contrary, they are representative of attitudes towards UA in the “post-pandemic period.” At the same time, we also believe that it is necessary to continue to carry out more in-depth studies to investigate how attitudes toward UA have changed before and after the pandemic.

Although the above issues remain, this study quantitatively evaluated the economic value of UA through a survey conducted in Shanghai, the largest city in China. It is important to further promote such research, generalize the findings of UA, and reflect them in policies through a broader survey which includes other urban areas.

## Ethical statement

All participants/patients (or their proxies) provided informed consent to participate in the study.

## Data availability

Data used in this study will be made available on request.

## CRediT authorship contribution statement

**Shengyi Du:** Writing – review & editing, Writing – original draft, Validation, Software, Resources, Project administration, Methodology, Investigation, Formal analysis, Data curation, Conceptualization. **Katsuya Tanaka:** Writing – review & editing, Writing – original draft, Validation, Software, Resources, Project administration, Methodology, Investigation, Funding acquisition, Formal analysis, Conceptualization.

## Declaration of competing interest

The authors declare that they have no known competing financial interests or personal relationships that could have appeared to influence the work reported in this paper.

## Footnotes

1 The destruction or covering of the ground by an impermeable material, which often affects fertile agricultural land, puts biodiversity at risk, increases the risk of flooding and water scarcity, and contributes to global warming.

2 Cities that do not belong to the highest-level regional administrative divisions, such as provinces and autonomous regions, but are placed at the same level as them and are directly under the jurisdiction of the central government. There are currently four cities in China: Beijing, Shanghai, Tianjin, and Chongqing.

3 Ctrip official website: https://www.ctrip.com/; Tongcheng Travel official website: https://www.ly.com/; Fliggy official website: https://www.fliggy.com/; Meituan official website: https://www.meituan.com/.

4 1 CNY is about 0.14 EUR (as of December 2022).

5 “Urban Land Use Classification and Planning and Construction Land Use Standards (GB50137-2011).” “Urban Green Space Classification Standards (CJJ/T85-2017).”.

6 The complete questionnaire (version 1 as an example) can be viewed through the following website: https://bit.ly/3y0BMww.

7 Wenjuanxing (Changsha Ranxing IT Ltd.) official website: https://www.wjx.cn.

8 Using the virtual valuation method, they surveyed 1005 taxpayers (aged 18+ and under 88 years) residing in Oslo to determine their annual willingness to pay for UA.

9 2021 data published on the official website of the National Bureau of Statistics of China: http://www.stats.gov.cn.

10 Under the guidance of the Department of Marketing and Informatization of the Ministry of Agriculture and Rural Development, Shanghai Jiaotong University carries out an indicator evaluation of the development level of modern agriculture in the nation's urban areas every two years and releases a report. Report details can be found on the official website of the Shanghai Municipal Commission of Agriculture and Rural Development: https://nyncw.sh.gov.cn/mtbd/20230302/a9527684fa2642cdb587d1a017734fe5.html.

11 The ML2 model has the smallest AIC.

12 This item is a general ticket for one adult. The price for weekdays is 435 CNY, and for holidays and public holidays, it is 545 CNY. Shanghai Disneyland official website: https://www.shanghaidisneyresort.com.

13 Food and beverages sold at the park's restaurants range in price from 60 to 300 CNY.

14 By matching and analyzing data held by the government and companies with the individual's reported information and behavioral history collected by the smart device, the individual's risk of infection will be divided into three levels: ‘green’ (low), ‘yellow’ (medium), and ‘red’ (high), with a barcode in each color. Along with the relaxation of the corona policy in China, it will no longer be necessary to inquire about health codes when entering or leaving public spaces (except in certain areas) as of December 13, 2022.

15 Circular of the Shanghai Municipal People's Government on the Issuance of Action Program for Promoting High-Quality Agricultural Development in Shanghai (2021–2025): https://www.shanghai.gov.cn/nw12344/20210112/0123cc48b40f403d8b787032cea9d194.html.
