# Supplementary material for: Residents' preferences for urban agriculture in Shanghai
Source: Heliyon. 2024 May 9;10(10):e30974. doi: 10.1016/j.heliyon.2024.e30974 (PMC11112317; doi:10.1016/j.heliyon.2024.e30974)
Supplement: Multimedia component 1 [file mmc1.pdf]

Questionnaire on Urban Agriculture and Disaster Prevention

This is a questionnaire about urban agriculture and disaster prevention, thank you for your cooperation and participation!

The questionnaire will be divided into four sections. In the first and second sections, we hope to learn about your attitudes and preferences towards urban agriculture and environmentally friendly production. In the third section, we will ask you about your experience in disaster prevention in the past 3 years. The fourth section is about your personal information. The fourth section contains some questions about your personal information.

This survey is anonymous. We assure you that all information collected will be used for academic research purposes only. Furthermore, we will ensure that your personal information will not be disclosed.

If you agree with the above content, please click "Next" to start the questionnaire.

Section 1

This is the first section of this questionnaire. We would like to know your level of knowledge and attitude towards urban agriculture.

1、 How much do you know about the following? Please select the one that best describes your situation.

	Very knowledgeable and familiar with the specifics	A little bit of knowledge, but not familiar with the details	Only heard the name	No understanding at all
Urban Agriculture				
Organic Production				
Green Production				

2、 Have you ever participated in urban agriculture?

☐ Yes ☐ No

3、 In 2021, what types of urban agriculture have you participated in *(Multiple choice)*? (*Those who chose "Yes" in the second question do not answer.*)

☐ Rooftop Farm ☐ Agri-tainment ☐ Country Parks ☐ Others( )

4、 Are you satisfied with the urban agriculture you have participated in the past? (*Those who chose "Yes" in the second question do not answer.*)

☐ Very satisfied ☐ Satisfied

☐ Relatively dissatisfied ☐ Very dissatisfied

5、 The ways you get information about urban agriculture are (*multiple choice*):

☐ Social media (Weibo, WeChat, TikTok, etc.) ☐ TV

☐ Newspapers and magazines ☐ Relatives

☐ Neighborhood, Resident Committee ☐ School, Workplace

☐ I saw it when passing by an urban farm by chance

☐ No information has been obtained about

6、 About the following, please select the one that best describes your situation.

	Very much in line	Comparable	Relatively inconsistent	Completely inconsistent
I take physical and mental health seriously.				
I attach great importance to food safety.				
I buy organic food regularly.				
Compared with ordinary products, I prefer to buy products that are environmentally friendly.				
I have experience in farming out of interest (e.g., planting on your balcony or yard).				

Do you know about urban agriculture?

Urban Agriculture (UA), as it is called, is a new type of agriculture that makes full use of unused space in the city for agricultural production. Some of them use the rooftops of neighborhoods or shopping malls or office buildings and are managed from the street, such as rooftop farms. Others are located in the suburbs, often in the form of "agri-farms", serving the surrounding and urban population. In addition to producing fresh produce, it also serves as a communal space and a refuge for the city's citizens. More importantly, urban agriculture also serves to improve the environment by improving air quality and increasing biodiversity in urban areas through the cultivation of various crops. At the same time, in some urban farms, citizens can experience plant cultivation or agricultural farming. Some urban farms have restaurants and stores, and some offer regular or irregular agricultural education programs. Urban agriculture serves the city and lives in symbiosis with it.

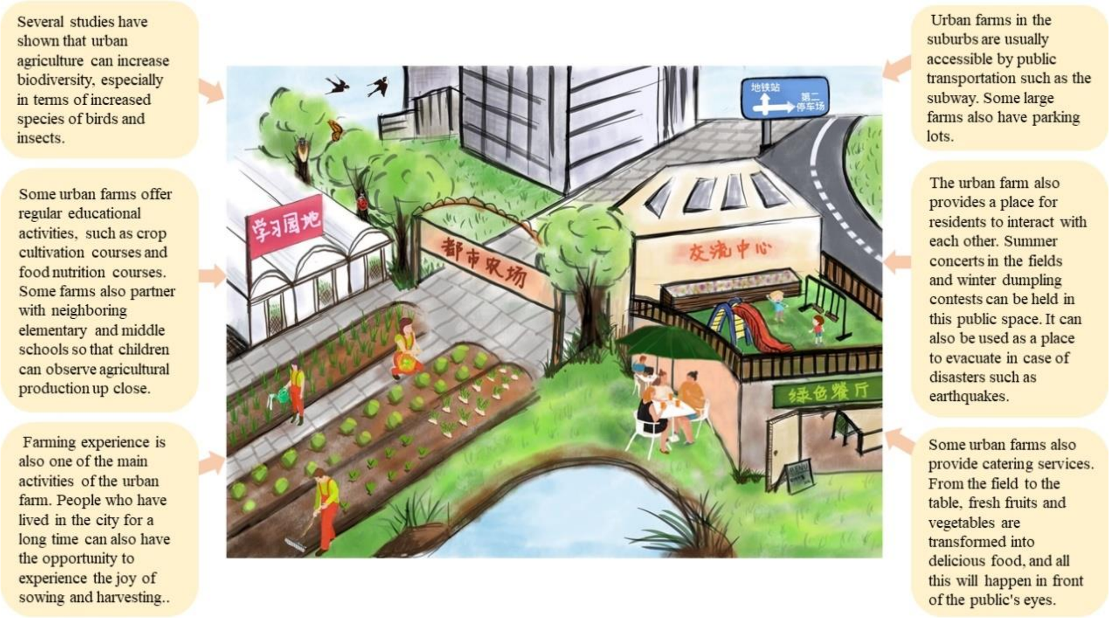

Figure 1. Explanation of UA and organic and green production (painted by the author).

Organic production and green production are common production methods in urban agriculture.

The definition of organic production in China is as follows: an agricultural production method that follows specific production principles, does not use organisms and their products obtained by genetic engineering, does not use chemically synthesized pesticides, fertilizers, growth regulators, feed additives and other substances, follows the laws of nature and ecological principles, coordinates the balance between cultivation and farming, and maintains a sustainable and stable production system. In short, organic production is a way of cultivation that does not use chemical fertilizers and pesticides, and is non-GMO and environmentally friendly.

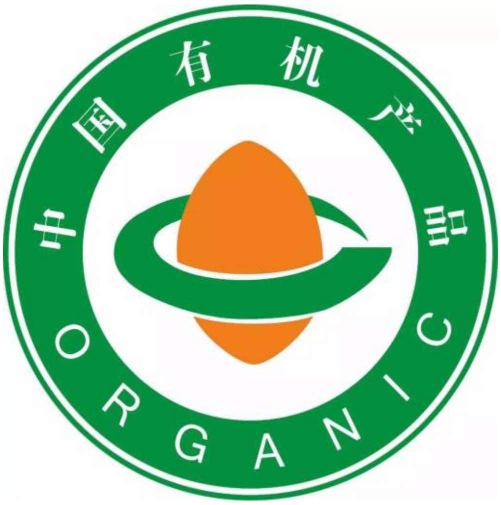

Figure 3. China Organic Product Mark

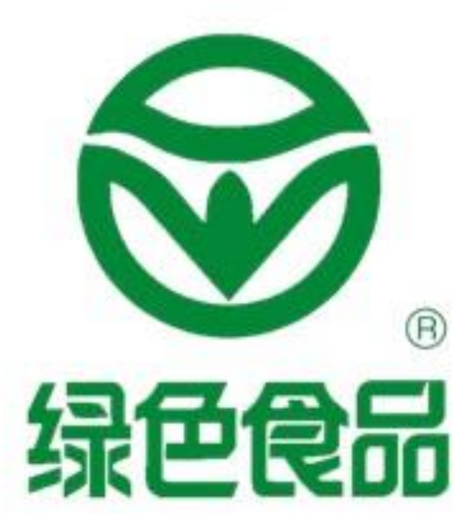

Figure 2. China Green Food Mark

Green production, on the other hand, is based on the premise of environmental protection and energy saving, allowing the use of some chemical fertilizers and pesticides in agricultural production that have a very low impact on human health and the environment.

Section 2

For the first 6 questions in this section, please allow us to give you a brief overview of how to answer them.

In each of the 6 sub-questions below, we will provide you with 2 models of urban agriculture, each with different characteristics, as shown in the chart below. We hope that you will be patient and look at all the attributes, consider the characteristics of both models and choose the one you would like to participate in. Of course, if you do not want to participate in either of the two models in this question, you can choose the last item "neither of the above", i.e. not to implement urban agriculture.

Please note that the 6 questions in this section are independent of each other and are not compared longitudinally.

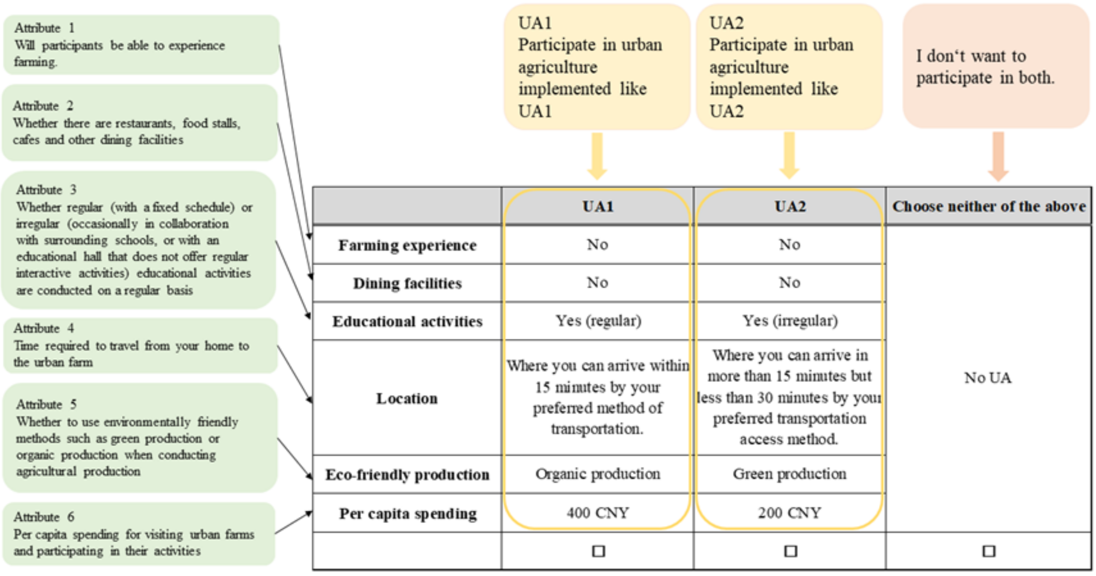

Figure 4. Example for a choice card.

7、 The following 2 kinds of urban agriculture are offered, each with different characteristics. Please consider the characteristics of each one and tell us which one you would prefer to participate in. If you do not want to participate in both, please select "Choose neither of the above" at the end.

	**UA1**	**UA2**	**Choose neither of the above**
**Farming experience**	Yes	No	No UA
**Dining facilities**	No	No	
**Educational activities**	No	Yes (irregular)	
**Location**	Where you can arrive within 15 minutes by your preferred method of transportation.	Where it takes more than 30 minutes to reach your destination by your preferred transportation method.	
**Eco-friendly production**	Green production	Organic production	
**Per capita spending**	100 CNY	100 CNY	
	☐	☐	☐

8、 The following 2 kinds of urban agriculture are offered, each with different characteristics. Please consider the characteristics of each one and tell us which one you would prefer to participate in. If you do not want to participate in both, please select "Choose neither of the above" at the end.

	**UA1**	**UA2**	**Choose neither of the above**
**Farming experience**	No	Yes	No UA
**Dining facilities**	Yes	Yes	
**Educational activities**	Yes (irregular)	Yes (rregular)	
**Location**	Where it takes more than 30 minutes to reach your destination by your preferred transportation method.	Where you can arrive in more than 15 minutes but less than 30 minutes by your preferred transportation access method.	
**Eco-friendly production**	Organic production	No	
**Per capita spending**	850 CNY	400 CNY	
	☐	☐	☐

9、 The following 2 kinds of urban agriculture are offered, each with different characteristics. Please consider the characteristics of each one and tell us which one you would prefer to participate in. If you do not want to participate in both, please select "Choose neither of the above" at the end.

	**UA1**	**UA2**	**Choose neither of the above**
**Farming experience**	Yes	No	No UA
**Dining facilities**	Yes	No	
**Educational activities**	Yes (rregular)	No	
**Location**	Where it takes more than 30 minutes to reach your destination by your preferred transportation method.	Where you can arrive in more than 15 minutes but less than 30 minutes by your preferred transportation access method.	
**Eco-friendly production**	Green production	Organic production	
**Per capita spending**	100 CNY	200 CNY	
	☐	☐	☐

10、The following 2 kinds of urban agriculture are offered, each with different characteristics. Please consider the characteristics of each one and tell us which one you would prefer to participate in. If you do not want to participate in both, please select "Choose neither of the above" at the end.

	**UA1**	**UA2**	**Choose neither of the above**
**Farming experience**	Yes	Yes	No UA
**Dining facilities**	No	Yes	
**Educational activities**	No	No	
**Location**	Where you can arrive within 15 minutes by your preferred method of transportation.	Where it takes more than 30 minutes to reach your destination by your preferred transportation method.	
**Eco-friendly production**	Green production	No	
**Per capita spending**	850 CNY	850 CNY	
	☐	☐	☐

11、The following 2 kinds of urban agriculture are offered, each with different characteristics. Please consider the characteristics of each one and tell us which one you would prefer to participate in. If you do not want to participate in both, please select "Choose neither of the above" at the end.

	UA1	UA2	Choose neither of the above
**Farming experience**	Yes	Yes	No UA
**Dining facilities**	Yes	Yes	
**Educational activities**	Yes (rregular)	Yes (irregular)	
**Location**	Where you can arrive within 15 minutes by your preferred method of transportation.	Where you can arrive in more than 15 minutes but less than 30 minutes by your preferred transportation access method.	
**Eco-friendly production**	Organic production	Green production	
**Per capita spending**	100 CNY	200 CNY	
	☐	☐	☐

12、The following 2 kinds of urban agriculture are offered, each with different characteristics. Please consider the characteristics of each one and tell us which one you would prefer to participate in. If you do not want to participate in both, please select "Choose neither of the above" at the end.

	UA1	UA2	Choose neither of the above
**Farming experience**	Yes	No	No UA
**Dining facilities**	No	Yes	
**Educational activities**	No	No	
**Location**	Where you can arrive within 15 minutes by your preferred method of transportation.	Where you can arrive within 15 minutes by your preferred method of transportation.	
**Eco-friendly production**	Green production	Green production	
**Per capita spending**	100 CNY	400 CNY	
	☐	☐	☐

13、About the following, please select the one that best describes your situation.

	Very much in line	Comparable	Relatively inconsistent	Completely inconsistent	Have no idea
I think it is important for agricultural products to be produced and sold locally.					
I think green production and organic production are very important.					
I think urban agriculture can feed food even during the COVID-19 pandemic.					
I think urban agriculture can be a recreational activity even during the COVID-19 pandemic.					
I agree that implementing urban agriculture can improve biodiversity.					
I agree that implementing urban agriculture can improve air quality.					
I think it is better to do agriculture in the countryside.					

Section 3

In this section, we will ask you about your past experiences with disaster preparedness and your attitudes toward disaster prevention.

14、Have you been affected by typhoon disasters in the past three years?

For example, Typhoon Lichma in 2019, Typhoon Hegbi in 2020, and Typhoon Smoky in 2021.

☐ Strongly affected

☐ Slightly affected

☐ Not affected although I was in Shanghai during that period

☐ Not affected because I was not in Shanghai during that period

15、Are you fully prepared for typhoon disasters that may occur in the future ?

☐ Very well prepared ☐ Well prepared

☐ Only a little bit of preparation ☐ Not prepared

16、The reasons why you are not fully prepared for typhoon disasters that may occur in the future are (*multiple choice*): (*Those who chose "Not prepared" in the previous question answer this question*)

☐ I don't have enough time.
☐ I don't have enough money.
☐ I don't know what disaster preparedness is.
☐ I think it is very unlikely that a typhoon will threaten me.
☐ I think typhoon preparedness is the responsibility of the government.

17、About disaster preparedness, please select the one that best describes your situation.

	Very much in line	Comparable	Relatively inconsistent	Completely inconsistent
I know the shelters in my community.				
I know where I live that is vulnerable to disasters.				
I spent time learning how to prepare for a disaster.				
In the event of a disaster, my family and I have plans for where to meet.				
I have water and emergency supplies in my house in case of a disaster.				
My family has an evacuation bag for evacuation.				
I participated in a community evacuation drill.				
I discuss disaster preparedness with people in my community.				
During a disaster, I know who in my community needs help.				
I downloaded the disaster alert app.				

18、The ways you get information about disaster are (*multiple choice*):

☐ Newspaper ☐ TV ☐ Radio

☐ Disaster alert app (Weather pass, etc.)

☐ Chatting with others ☐ Social media (WeChat, Weibo, etc.)

19、Do you trust the following?

	Completely trust	Comparing trust	Slightly distrust	Total distrust	Have no idea
Passers-by					
Neighbor					
Good friend					
Resident Committee					
Social media (Weibo, WeChat, etc.)					
Mass media (TV shows, radio, search engines, etc.)					

Section 4

This is the last section of this questionnaire and we would like to know something about you personally.

20、Your gender is:

☐ male ☐ female

21、Your age is:

☐ 20-29 years old ☐ 30-39 years old ☐ 40-49 years old

☐ 50-59 years old ☐ 60 and over

22、Your monthly household income (including investment income such as stocks or funds, house rent, etc.) is approximately:

☐ 3,000 CNY and below

☐ 3,001 CNY - 6,000 CNY

☐ 6,001 CNY - 9,000 CNY

☐ 9,001 CNY - 12,000 CNY

☐ 12,001 CNY - 15,000 CNY

☐ 15,001 CNY - 18,000 CNY

☐ 18,001 CNY - 21,000 CNY

☐ 21,001 CNY - 24,000 CNY

☐ 24,001 CNY - 27,000 CNY

☐ 27,001 CNY - 30,000 CNY

☐ 30,001 CNY and above

23、Your highest education is:

☐ Elementary school ☐Junior high school

☐ High school or Vocational school or Technical secondary school

☐ Short-cycle Courses ☐Bachelor ☐ Master and above

24、Which district in Shanghai do you live in?

☐ Huangpu ☐Xuhui ☐ Changning ☐ Jing'an ☐ Putuo ☐ Hongkou

☐ Yangpu ☐ Minhang ☐ Baoshan ☐ Jiading ☐ Pudong New Area

☐ Jinshan ☐ Songjiang ☐ Qingpu ☐ Fengxian ☐ Chongming ☐ Oher

25、When did you start living in Shanghai?

☐ since birth ☐ Before 1962

☐ 1962... ☐ 2020 (Click on the drop-down to select)

☐ After 2020

26、Your marital status is:

☐ Single ☐ Married

27、Do you have children under the age of 15?

☐ Yes (I have children under the age of 5.)

☐ Yes (I have children between the ages of 6-15.)

☐ No (I have children over the age of 16.)

☐ No (I don't have children.)

28、Do you have farming (including horticulture) or farming-related experience?

☐ Yes ☐ No

29、Are you willing to take the risk, even if you have the opportunity? Or prefer to be risk-averse?

Avoid risk									Take the risk
1	2	3	4	5	6	7	8	9	10
☐	☐	☐	☐	☐	☐	☐	☐	☐	☐

30、Finally, can you share your happiness with us?

☐ I feel very happy. ☐ I feel relatively happy.

☐ I feel a little bit unhappy. ☐ I feel very unhappy.
